# Meaning in Life Among Aged People: A Qualitative Study of an Institutionalized Elderly Sample

**DOI:** 10.3390/ejihpe15060091

**Published:** 2025-05-22

**Authors:** Lăcrămioara Cojocaru, Camelia Soponaru, Daniela Muntele-Hendreș, Ciprian Ceobanu

**Affiliations:** Faculty of Psichology and Educational Sciences, Alexandru Ioan Cuza University, 700554 Iaşi, Romania; lacramioara.cojocaru@uaic.ro (L.C.); camelia.soponaru@uaic.ro (C.S.); hendres@uaic.ro (D.M.-H.)

**Keywords:** old adults, qualitative research, religiosity/spirituality, intergenerational values transmission, interpersonal relationships

## Abstract

As individuals grow older, they experience notable shifts in their health conditions, social positions, and personal identity. Studies show that finding meaning in life correlates with healthier lifestyle choices and enhanced overall well-being. This sense of meaning functions as a protective factor against life’s challenges, strengthening resilience during later years. The research utilized thematic analysis within a qualitative methodology to investigate the subjective sources of meaning among elderly people living in Romanian institutional settings. The analysis revealed several interconnected themes. Religiosity and spirituality emerged as a fundamental anchor, with participants often mentioning God, Divinity, and Higher Power in their narratives. They typically conceptualized life as a sacred gift and emphasized the importance of religious practices such as prayer and church attendance as essential to their sense of purpose. The intergenerational transmission of values also proved significant, with elderly individuals finding meaning in passing down moral and spiritual principles to younger family members, viewing this as their enduring legacy. Interpersonal connections played a crucial role as well, with quality relationships with family and friends fostering a sense of belonging, while broken relationships or isolation negatively impacted well-being. Daily life objectives, including short-term goals like maintaining routines or anticipating family visits, along with preserving functional independence, provided structure and immediate purpose for many participants. The research distinguished between religiosity and spirituality as related but separate concepts: while formal religious rituals offered community-based meaning, those less engaged with organized religion discovered transcendence through personal spiritual experiences such as connecting with nature. This study highlights cultural specificities, particularly how Orthodox Christian traditions shape existential narratives among Romanian elderly, and proposes practical interventions for retirement facilities to incorporate activities focused on spiritual engagement, relationship building, and opportunities for elders to share their wisdom with younger generations.

## 1. Introduction

Older adults derive meaning from various components, including purpose, moral worth, self-worth, control, coherence, excitement, and connectedness. These elements are crucial in shaping life narratives and providing a framework for understanding life experiences in older age (Derkx et al., 2020). Meaning in life refers to the significance and purpose individuals attribute to their existence. It encompasses the beliefs, values, and goals that give life direction and coherence. Research has shown that having a sense of meaning is associated with various positive outcomes, including better mental and physical health, enhanced well-being, and improved coping mechanisms in the face of stressors (Schnell, 2009, 2020).

The influence of these outcomes on the meaning in life was found to be significant. When referring to health and well-being, studies indicate that individuals who perceive their lives as meaningful tend to engage in healthier behaviors and experience better overall health (Bratt & Fagerström, 2023). Meaningfulness has been linked to lower levels of stress and better recovery from illness (Steptoe & Fancourt, 2019) and was found to be a protective factor against cognitive decline (Kim et al., 2019). Meaning acts as a moderator in the relationship between stressors and distress. Individuals with a strong sense of meaning are better equipped to handle life’s challenges, experiencing less severe suffering during difficult times (Mäkikangas & Kinnunen, 2003).

The individual’s need to search for meaning in life is a phenomenon known and highlighted in the literature within humanistic-existentialist approaches as found in the works of authors such as Frankl, Maslow, and Antonovsky.

The search for meaning is the primary motivation in one’s life and not a ‘secondary rationalization’ of instinctual drives, as Viktor Frankl (2000) wrote in his fundamental book *Man’s Search for Meaning*. This task of finding meaning in life does not diminish with age, and in fact, its pursuit can become more pronounced as life goes by. Among older populations, the concept of meaning of life can be interpreted from different theoretical perspectives.

Among these, Antonovsky’s theory on the sense of coherence might be considered a cornerstone in understanding how individuals find meaning in life, particularly in the context of health and well-being (Eriksson & Lindström, 2005). Meaningfulness is a multidimensional construct that is applicable across different cultures and life stages, providing a framework for understanding how individuals find meaning and manage stress in their lives. The integration of meaningfulness into health models highlights its importance in both psychological and physical health outcomes (Eriksson & Mittelmark, 2017). Although considerable research has explored sources of meaning in life among older adults internationally, much of this work has been conducted in Western contexts, with relatively little attention paid to Eastern European cultural settings. Romania—with its distinct socio-historical evolution, religious traditions, and social structures—offers a unique context for investigating how older adults construct meaning in life.

## 2. Theoretical Background

Meaning in life is often understood as a multidimensional construct (Schnell, 2009, 2020; Schnell & Becker, 2006), which means it encompasses various components or dimensions that contribute to an individual’s overall sense of meaning. This complexity reflects the diverse ways in which people find significance in their lives. The key features typically associated with the construct of meaning in life are as follows:

Purpose in life is defined by having core goals, aims, and a sense of direction. It involves setting and pursuing meaningful goals that align with one’s values and aspirations (Martela & Steger, 2016). A strong sense of purpose is associated with numerous adaptive benefits, including improved mood, motivation, and goal achievement. It also enhances cognitive function and supports better cognitive aging outcomes (Sutin, 2023). Individuals with a clear sense of purpose are more likely to set and achieve goals, which in turn reinforce their sense of meaning and direction in life (Owen et al., 2022).

Significance denotes the belief that one’s life possesses inherent value and contributes meaningfully within a broader social or existential context. It encompasses a sense of personal worth and the perception that one’s existence holds relevance both for oneself and for others. Meaning in life often arises from activities that are grounded in love for objectively valuable objects, rather than from a mere interest in meaningfulness itself. However, what constitutes a “worthy” object is subjective and cannot be determined from a completely neutral standpoint (Wolf, 2014). Research suggests that meaning in life includes feelings of coherence, purpose, and external value, such as significance or mattering. Internal value, or one’s perceived internal worth, is also considered a fundamental component of meaning in life (Li et al., 2020).

Coherence, in the context of life experiences, refers to the ability to perceive life as comprehensible, manageable, and meaningful. These components influence how individuals perceive their quality of life over time. Comprehensibility affects the importance of present and past events, while manageability relates to the control over life events. Studies have shown that individuals exposed to organized patterns report higher levels of meaning compared to those exposed to random patterns (Heintzelman et al., 2013). Other research suggests that life experiences such as consistency, load balance, migration, and integration experiences play a role in influencing life’s sense of coherence (Slootjes et al., 2017). In this respect, individuals with a strong sense of coherence are better equipped to integrate their experiences into a meaningful narrative, which is linked to improved psychological well-being and quality of life. While coherence is a component of meaning in life, existential mattering (feeling significant) is a stronger precursor to judgments of life meaning. This suggests that feeling significant may be more crucial than coherence or purpose alone (Costin & Vignoles, 2020).

The belonging dimension of meaning in life emphasizes the importance of social connections and relationships, which can significantly enhance one’s sense of meaning. Different studies have found a strong positive correlation between the sense of belonging and the perceived meaningfulness of life, across various methodologies, including correlational, longitudinal, and experimental studies (Zhang et al., 2018; Lambert et al., 2013). Threats to belongingness can reduce the importance of social relationships as a source of meaning in life, but individuals may compensate by seeking autonomy (Zhang et al., 2018). Another significant observation is related to the bidirectional relationship between social connectedness and meaning in life (O’Rourke & Sidani, 2017). Among different types of connectedness, collective connectedness is particularly associated with meaning in life (Stavrova & Luhmann, 2016). This bidirectional relationship highlights the importance of fostering social connections to enhance one’s sense of meaning in life.

Transcendence involves experiences that extend beyond the self, such as spirituality, connection to nature, and activities that evoke awe and wonder. This concept is explored through various lenses, including gero-transcendence, self-transcendence, and collective transcendence. Transcendence is considered a crucial aspect of successful aging, providing a framework for interventions that enhance life satisfaction and well-being among older adults (McCarthy & Bockweg, 2013).

While religiosity and spirituality are often intertwined, research suggests they serve distinct psychological functions. Among older adults with low institutional religiosity, spirituality (e.g., connection to nature, cosmic transcendence) may compensate for the absence of structured religious frameworks by providing alternative avenues for meaning-making. This pattern is particularly pronounced in adults over 75, who may prioritize personal spiritual experiences over dogma as they confront mortality. The apparent contradiction—that spirituality matters more for the less religious—reflects a broader existential adaptation: when traditional religiosity wanes, transcendent beliefs persist as a resilient source of coherence (Braam et al., 2006). Other studies indicate that people perceive meaning in life more through self-transcendence (benefits to society) than self-enhancement (benefits to the self). This contrasts with happiness judgments, where self-enhancement plays a more significant role (Huang & Yang, 2022). The concept of self-transcendence, as described by Viktor Frankl, is seen as essential for well-being, emphasizing a shift from materialistic pursuits to spiritual fulfillment. This approach is linked to better mental health and is particularly beneficial for those facing chronic illness or end-of-life situations (Wong, 2016).

Self-transcendence is also explained by Tatjana Schnell (2012), as vertical—through explicit religiosity and spirituality—and horizontal—through self-knowledge, social commitment, as well as health, generativity, and union with nature. This means that religious practices and spiritual beliefs are important sources of meaning for the elderly, as well as personal growth, relatedness, and generativity as a part of social commitment.

All the key factors depicted above indicate that meaning in life among elderly people is influenced in various ways. Research consistently shows that these factors significantly contribute to older people’s mental health and overall well-being. Among these, closely linked to the old person’s meaning in life are their levels of religiosity and spirituality and the quality of their relationships.

Understanding meaning in life as a multidimensional construct allows for a more nuanced approach to research and interventions aimed at enhancing well-being. It recognizes that individuals may derive meaning from different sources and that these sources can interact in complex ways to shape their overall sense of purpose and significance in life.

## 3. Methods

### 3.1. Research Question

It is obvious that sources of meaning in life differ with age. For young adults, the main sources of meaning in life are considered to be relationships, especially family connections (Debats, 1999), personal and professional goals (Bhattacharya, 2011), and parental influence; young adults with supportive parental backgrounds tend to have a stronger sense of meaning in life (Kealy et al., 2020).

In middle-aged adults, the meaning in life is influenced by various sources and factors, which are essential to their psychological well-being and life satisfaction. Key sources of meaning include relationships and health (Karwetzky et al., 2021; Debats, 1999), spirituality and intrinsic religious orientation (Hee & Nam, 2018), as well as personal growth (Fave et al., 2013; Dolberg & Ayalon, 2018). Also, factors such as social support, self-esteem, and stress play significant roles (Hee & Nam, 2018; Chang & Sohn, 2017).

Older adults often find meaning in life through various sources, which significantly impact their well-being, health, and quality of life. For older people, family and close relationships are consistently highlighted as primary sources of meaning (Hupkens et al., 2018; Zhou et al., 2019). Many older adults find meaning through spirituality and religious practices (Jonsén et al., 2015). Engaging in unselfish activities that promote personal growth and contributing to future generations (Sørensen et al., 2021), as well as maintaining good health, having a sense of life, and security are crucial sources of meaning in life for older adults (Hoeyberghs et al., 2019; Zhou et al., 2023). Finally, some older adults find meaning in their connection to nature and their role as a link between generations, which involves passing on traditions and values (Jonsén et al., 2015).

Starting from all these premises, our research question is: What are the main sources of meaning in life for elderly adults, and what are the main features of meaning in life within the research group?

### 3.2. Methodology

This study employed a qualitative–exploratory research design, guided by a phenomenological approach, aiming to explore the subjective sources of meaning in life among institutionalized elderly individuals. Because of the small number of participants, the results cannot be generalized to a larger population. Qualitative research seeks to understand social phenomena by capturing participants’ perspectives and interpreting them within their relational and cultural contexts (Rossman & Rallis, 2016; Bradbury-Jones et al., 2017).

The research began in January 2024 and involved the development of a semi-structured interview guide, constructed based on the main orientations identified in the existing literature. The guide included open-ended questions exploring existential meanings, interpersonal relationships, spirituality, and intergenerational value transmission, as well as several dichotomous and demographic questions. The study protocol was reviewed and approved by the Research Ethics Committee of the affiliated academic institution. The study included 25 institutionalized residents (11 women and 14 men), aged between 65 and 93 years. The participants were aged as follows: 65–74 years—9, 75–84 years—7, 85–93 years—9. Educational backgrounds varied: the majority had completed secondary education (high school or lower), while a smaller number had attended higher education institutions. Marital status was also diverse: several were widowed or divorced, while others remained married. Participants had a range of former occupations, including skilled trades (e.g., foreman, electrician), education, law enforcement, agriculture, and domestic work. This demographic diversity provided a rich context for exploring subjective sources of meaning in later life. Each interview lasted approximately 60 min and was audio-recorded with the participant’s permission. The recordings were securely stored on encrypted drives. Interviews were conducted by L.C. and C.S., both trained psychologists, under the coordination of L.C. Inclusion criteria were: institutionalization, age 65+, cognitive ability to sustain dialogue, and informed consent. Those with significant cognitive impairment were excluded. Audio recordings were transcribed via Transkriptor© v.1 and manually verified for accuracy by the research team (L.C., C.S., D.M.H., and C.C.) prior to thematic analysis.

Following data collection, the interviews were transcribed verbatim using Transkriptor© v.1 software and manually corrected for accuracy. Translation from Romanian to English for analytic and reporting purposes was conducted by two independent bilingual researchers. A third bilingual reviewer verified the translations to ensure semantic and contextual fidelity. For data analysis, we employed thematic analysis as described by Braun and Clarke (2006), following a rigorous six-phase process: familiarization with data, initial coding, theme searching, theme reviewing, theme defining and naming, and report production. Initially, NVivo 14© software was used for preliminary coding; however, due to the limitations of semantic recognition in Romanian, manual thematic coding was subsequently undertaken.

L.C. and C.S. independently coded a subset of transcripts, then discussed initial codes with D.M.H. and C.C. to refine the thematic structure. Themes were derived inductively, reflecting patterns across the dataset, and involved triangulation between two independent researchers to enhance analytic credibility. Five major patterns emerged from the data: (1) principles of life, (2) spirituality, (3) religiosity, (4) subjective well-being, and (5) meaning of life through objectives. The thematic analysis enabled us to capture both shared and divergent experiences, linked to participants’ demographic profiles and personal life histories. In summary, the methodological design was carefully structured to ensure methodological rigor, transparency, and adherence to COREQ guidelines (Appendix A), allowing an in-depth exploration of the subjective meaning in life among elderly individuals in an institutional setting.

## 4. Results

**Figure 1:**
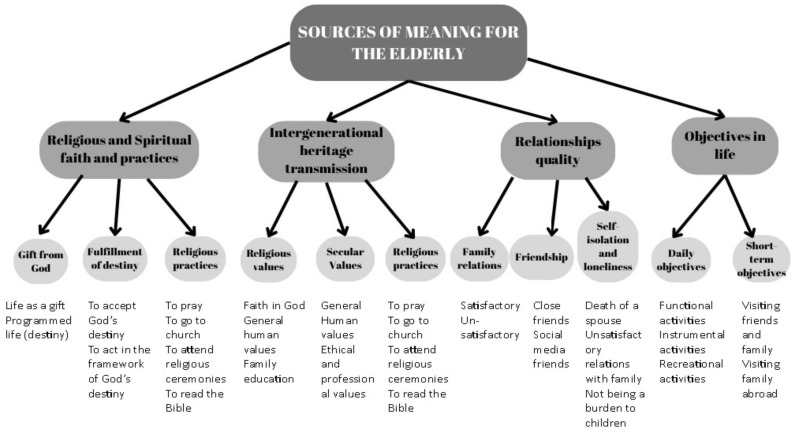
The theme structure: “sources of meaning for the elderly”.

Four codes were identified following the analysis of the interviews carried out with the participants (institutionalized elderly), which allowed for the integration of similar themes under one “umbrella” (Figure 1). The “sources of meaning for the elderly” codebook encompasses the following general themes:

- Religious and spiritual faith and practices describe the elderly’s convictions about life as a “gift” from God, in the form of an individual destiny, offered by Divinity. These are accompanied by religious practices that anchor faith in a behavioral manner, both at the individual and community levels.
- Intergenerational heritage transmission describes the fact that elderly people consider the spiritual legacy they leave behind to be very important; the legacy is described in terms of moral values and includes, in addition to religious values, secular values, carried out through parental and institutional education (school or workplace). Again, religious practices appear as a sub-theme, representing a traditional value that should be preserved and transmitted to the younger generation, as a complementary source of meaning.
- The quality of relationships is described as positive or negative, and it is linked to the present moment or to previous life experiences, regarding family or friends. The quality of the family relationships influences the tendency toward self-isolation, accompanied by the feeling of loneliness.
- Life objectives describe daily routines and short-term plans (1–3 months, up to a year) and include recreational activities, intentions to visit children who live and work abroad, and tourist visits.

With the exception of the intergenerational heritage transmission theme and the unsatisfactory relationships within the family context, to which they refer in the past tense, the remaining themes are derived from their present experiences and convictions, formulated in the present tense.

**Figure 2:**
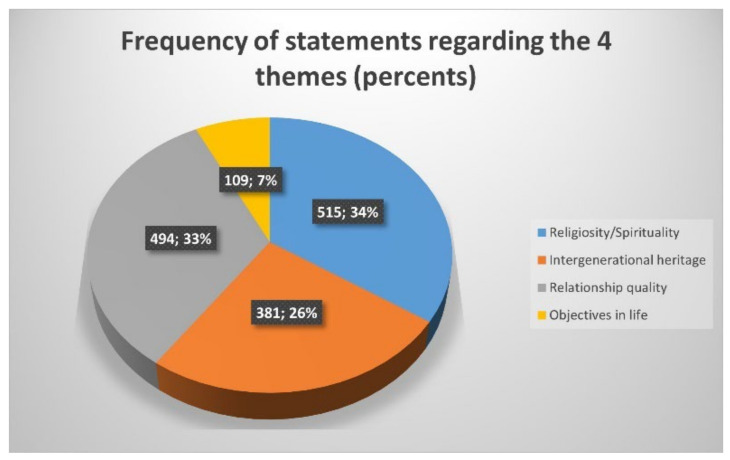
Frequency of statements regarding the four themes.

To provide a clearer picture of the relative significance of these themes within the study group, we summarized the number of statements for each theme in the study group (Figure 2). These proportions suggest the prominence of transcendental and relational dimensions in constructing meaning in life among the participants.

### 4.1. Religiosity and Spirituality as Foundations for Meaning in Life

Religiosity and spirituality emerged as the primary frameworks through which participants structured and interpreted their existence, offering coherence, purpose, and resilience in the face of life’s challenges. In general, spirituality and religiosity, while often interconnected (Agniete et al., 2021), refer to different concepts. While spirituality was described as an individual search for existential connection and personal transcendence, religiosity represented adherence to communal beliefs and rituals. However, for the participants, these two dimensions were deeply intertwined, jointly shaping their understanding of life’s meaning. Faith was recurrently articulated as the source of existential significance, grounding participants in a larger, transcendent order: “I believe in God; faith is very important, without faith there is no meaning in life.” (I67, female, late 60s).

Such statements illustrate that for these individuals, faith is not merely an abstract belief but the very medium through which life acquires meaning, providing assurance that existence itself is part of a divine design.

The concept of vertical transcendence (Schnell, 2012)—striving toward connection with a higher power—was deeply embedded in participants’ narratives. Ritual practices (prayer, worship, fasting) were not perceived solely as obligations, but as acts of reaffirmation of their connection to a meaningful, sacred order: “In the morning, we worship God… it keeps me grounded and thankful every day.” (I65, male, mid 80s). Through these actions, participants actively anchored their daily experiences within a spiritual framework, thereby reinforcing the perceived meaningfulness of their lives.

Destiny and divine will were also key components of meaning-making. Participants conceptualized life events—both positive and negative—as parts of a divinely orchestrated plan, which they were called to accept and fulfill: “It is destiny up to a certain point.” (I82, female, early 70s); “Both good and bad come from God, and we must respect Him.” (I65, male, mid 80s).

Acceptance of destiny thus emerged as an existential orientation that infused suffering and uncertainty with meaning, transforming adversity into opportunities for reaffirming faith and moral growth.

Importantly, the internalization of religious values shaped participants’ moral decision-making, reinforcing life’s coherence and sense of purpose: “When I do something, I think if it’s right or wrong—it’s doing God’s will.” (I62, female, late 60s).

Through aligning personal choices with divine principles, participants embedded moral worth into their daily lives, thus actively constructing meaning in accordance with a higher ethical framework. Adversity—such as illness or family hardships—often prompted a deepened reengagement with faith. Participants described experiences where suffering became a catalyst for reaffirming or transforming their relationship with God: “My son got sick and from that moment I stopped swearing.” (I66, male, early 70s); “[Now] I immediately come to terms with the idea that this is the way it was meant to be.” (I66, male, early 70s).

This process illustrates how faith was dynamically reconstructed across the lifespan, adapting to existential crises and providing tools for emotional regulation and acceptance. Moreover, meaning was not only found in transcendent beliefs but also in concrete life achievements interpreted through a religious lens—procreation, ethical family life, community contribution, and personal integrity: “I brought up a child.” (I62, female, late 60s); “Spouses must love and understand each other and raise their children calmly.” (I78, female, mid 80s); “You must think about what you are doing.” (I81, male, mid 80s).

Through these domains, participants expressed a profound sense that everyday actions were spiritually significant, contributing cumulatively to the meaningfulness of their existence.

Engagement in religious practices—attending church, praying, maintaining sacred objects—was seen as a continuous act of reaffirmation and anchoring of meaning, especially in older age: “In my house, you will find a cross consecrated by the priest and everything.” (I77, female, early 80s); “Go to church, take communion.” (I67, female, late 60s).

However, some participants also demonstrated a reflective and individualized stance toward religious observances, suggesting a nuanced, critical engagement with traditional practices: “No one can force me to worship Saint Parascheva only on the feast day. I can visit anytime.” (I70, male, early 80s).

This flexibility highlights that spiritual meaning-making was not static but evolved with personal experiences, convictions, and critical reflection, emphasizing participants’ agency in shaping their religious lives.

In summary, religiosity and spirituality in later life served as multi-layered frameworks of meaning-making, combining belief, practice, moral action, and existential acceptance into a dynamic, resilient structure that sustained participants’ sense of purpose, coherence, and fulfillment.

### 4.2. Intergenerational Heritage Transmission

Intergenerational heritage transmission plays a crucial role in shaping the meaning of life for older adults. This process involves the exchange of values, traditions, and knowledge between generations, which can significantly impact the well-being and life satisfaction of older individuals. Intergenerational interactions have been shown to positively affect older adults’ physical and mental health. These interactions can reduce feelings of loneliness and stress, provide a greater sense of purpose, and enhance life satisfaction. They also foster appreciation for generational differences and strengthen social connections, which can improve mental and physical health and reduce the risk of depression (Zhong et al., 2020; Krzeczkowska et al., 2021).

The values received from elders are often reflected in the values that individuals pass on to their descendants, suggesting a significant familial influence (Albanese et al., 2014).

It has been observed that there are two main bodies of principles and values that are transmitted from the older generation to the younger ones: (i) *religious beliefs* that are considered to be parts of the individual and social identity and (ii) *secular principles and values* that serve as sources for meaning in life. The axiological orientation has become an important part of defining meaning in life for this age group. As mentioned earlier, these values have served as both sources of meaning in life and as axiological objects that have been transmitted to the younger generation.

The early years of a child’s life are pivotal for religious education, with family members, particularly older adults, playing a significant role in this process. Older adults frequently become key figures in nurturing children’s spirituality, providing support and guidance in religious education activities (Allana et al., 2017).

The involvement of grandparents in religious education is seen as enabling and supportive, helping to embed spirituality in young children and fostering a conducive environment for faith development.

The transmission of religious values (which are closely related to religious covenants) is another element that can be noted in the context of this analysis: “And I think another principle (that can be transmitted) is the relationship with divinity.” (I66, male, early 70s); “The values that come through (the philosophy of religious life) religion, religion doesn’t teach us anything wrong.” (I84, male, early 80s).

The transmission of religious values within families is a pivotal process, with grandparents playing a significant role as educators and role models. Despite the challenges posed by time constraints and cultural pluralism, effective strategies, such as family involvement and integrated educational approaches, have been shown to enhance the transmission of religious and moral values to the next generation. Addressing these challenges is imperative for maintaining the impact of family education on children’s spiritual and moral development: “Reaching the stage where I had to realize that there is divinity, telling my granddaughters to develop (faith).” (I67, female, late 60s); “I never had an in-depth discussion with my daughter or my son about the church, about God, about the spirit. That would be part of the spiritual heritage.” (I70, male, early 80s); “Because I have two children left. Let them also follow, let them believe in the good Lord as we believe.” (I73, female, late 60s); “A spiritual inheritance is a spiritual education. Love of parents, faith, love of friends, of children, of family.” (I83, male, mid 80s).

Religion significantly influences human values. The transmission of these values occurs through familial and societal structures, impacting individual and collective behaviors: “The greatest values are trust, kindness, sincerity, love. […] This code of moral values comes through religion.” (I82, female, early 70s).

Grandparents provide both emotional and material support, which is crucial for the transmission of family legacies, including secular values such as education, solidarity, and order (Schuler & De Souza Brito Dias, 2019). Their influence is shaped by family dynamics, cultural contexts, and the quality of their relationships with grandchildren: “Friendship and honesty are the guiding principles of my life.” (I74, male, early 70s); “Respect for people. Respect for laws for customs, for unwritten laws.” (I84, male, early 80s); “Something that people leave behind that is not material. For example, (life) lessons.” (I87, male, early 80s); “Spiritual heritage (depends) on the family. It is about the transmitted values through family education.” (I82, female, early 70s); “A way of behavior, the ABC of good behavior.” (I81, male, mid 80s).

General human values were also listed as meaning-making for the older people in the study group. They often rely on basic values such as dignity, honesty, and respect for tradition and values. These values provide a sense of continuity and integrity: “Let something be seen behind us, that something of value remains.” (I64, female, mid 80s); “Justice is a principle that has guided me throughout my life.” (I72, male, early 70s); “I am an honest person. I am guided by truth.” (I73, female, late 60s); “In general, I was a man of moderation. Being good, being kind, doing my duty.” (I86, male, mid 80s); “To be correct, a man of my word.” (I126, male, early 90s); “Love and respect for parents, love for country, and love of humankind.” (I81, male, mid 80s); “My guiding principles in life are punctuality and discipline.” (I75, male, mid 80s); “…fairness, perseverance” (I67, female, late 60s).

The values related to ethical and professional issues, which have previously and continue to provide meaning to the lives of older adults: “I would put first work ethic. Honesty, humanity. To have as much knowledge as possible, so self-development.” (I66, male, early 70s); “My profession, my work, honest and loyal to your friends, to everybody.” (I71, female, late 70s).

Older adults often experience increased religiosity as they age, which can influence their role in religious transmission. Their religious biographies, shaped by personal and societal changes, contribute to a flexible and open approach to religious discussions within families, facilitating successful transmission across generations (Spannari et al., 2021). The religious intensity and practices of parents, including older adults, have a lasting impact on their children’s religiosity. This influence is often mediated by early and midlife religious experiences, creating a momentum that carries into old age (Silverstein et al., 2022). Although religious practices are mentioned as creators of meaning, it should be noted that they represent a double source of meaning, both as a moral duty and as an anchor for fixing, preserving and transmitting faith and religious heritage: “In the morning, we worship God.” (I65, male, mid 80s); “I pray to God … give them (my boy and girl) good health.” (I70, male, early 80s).

### 4.3. The Quality of Relationships

The self-assessment component of the interview, which was designed to evaluate the current state of well-being among the elderly, offered valuable insights into the quality of family relationships and the presence of significant others in their lives. These relationships appear to be a source of meaning and a life objective, both of which are closely tied to religious belief and practice. Regular interactions with family, friends, and religious communities enhance the sense of belonging and contentment, contributing to a meaningful life (De Oliveira & De Oliva Menezes, 2018). The quality of social relationships, including support from religious congregations, is linked to better life satisfaction and quality of life in the elderly (Gallardo-Peralta, 2017). Older adults rely on family and friends for emotional, instrumental, and financial support. Those with strong ties to both groups are generally more socially and psychologically well adjusted (Adams & Blieszner, 1995). Social engagement (in-person interactions and group engagements), including interactions with family and friends, helps satisfy the need to belong and reduces feelings of loneliness (Tomstad et al., 2024). It is noticeable that the quality of relationships, particularly with friends, is a stronger predictor of life satisfaction than the frequency of contact. Friendships often provide more positive and meaningful interactions compared to family relationships (Larson et al., 1986; O’Connor, 1995). Interactions with friends are associated with increased positive affect and life satisfaction and decreased negative impact, while family interactions can increase both positive and negative affect, highlighting the complex dynamics of family relationships (Huxhold et al., 2014; Ng et al., 2020).

“(The meaning of life is given by relationships, only if human) makes himself useful to the community, to other people.” (I84, male, early 80s). It has been suggested that socializing can be a source of meaning: “I have very good relationships, relationships with friends and family… yes, relationships give meaning to life.” (I66, male, early 70s).

Engaging in social activities and leveraging technology to maintain connections can further support these relational aspects (Saarelainen et al., 2020). These relationships provide emotional support, reduce feelings of isolation, and enhance the overall quality of life: ”My mom lived with my dad until they were 73 years old. When he died, after 3 years, she withdrew completely.” (I62, female, late 60s); “(It’s important) to have your grandchildren visiting.” (I74, male, early 70s); “Relationships give meaning” (I83, male, mid 80s).

Family relationships significantly influence the meaning of life and well-being in older adults. The quality of relationships enhances quality of life and raises the well-being of the elderly person (Saarelainen et al., 2020). Positive relationships enhance life satisfaction and mental health, while negative interactions can detract from these outcomes. Addressing both positive and negative aspects of these relationships is crucial for supporting older adults in finding meaning and improving their quality of life: “If there would be no meaning in people working together, there would be no (quality) life, there would be no meaning in life they wouldn’t be able to create things.” (I67, female, late 60s).

Satisfactory family relationships increase life satisfaction: “You also have to match yourself with the person you’re in a relationship with, so that you can talk about the same fear. And you can create a thing.” (I63, female, early 70s).

While the interviews were conducted in the present tense, the elderly preferred to answer certain topics in the past tense. In order to maintain discretion and motivation to participate, we did not delve deeper into the interview but instead focused on extracting the themes from the life story that the elderly felt comfortable sharing: “It was very good how I programmed (life) him (my husband) and how I lived with him for so many years and had (together) three children, three boys, how I brought them up, I also did (work) in the house and worked, and I sent (the children) to school.” (I79, female, mid 80s); “I understand that a fulfilled life means a fulfilled life for others in the family.” (I80, male, mid 70s).

Unsatisfactory family relationships can significantly undermine the sense of meaning in life for older adults, primarily through increased depression and reduced quality of life. These effects are compounded by relational gaps and negative interactions, highlighting the importance of fostering positive family dynamics to support the well-being of older adults: ”The only thing I would like is to have a good relationship with my daughter-in-law (she won’t let me visit my son and grandchildren).” (I62, female, late 60s); “Deep relationships (give meaning to life)… my wife wouldn’t let me talk to my maiden until I was 18. I have tried, I have tried to get close to someone, but everything is on the surface, we just see each other.” (I72, male, early 70s); “It makes me sad, the relationship with my wife. I don’t hate her… she says it’s all my fault.” (I86, male, mid 80s); “I haven’t been in touch with my boys. They are in France. They only came to Romania to pay their taxes and for other things, often they didn’t even come to see me. They don’t even know I’m in the foster house.” (I74, male, early 70s).

Engaging in activities with peers and maintaining friendships can bring joy and a sense of connectedness, which are vital for meaning in life (Saarelainen et al., 2020). Both subjective (perceived) and objective (actual) social supports are linked to higher life satisfaction and health in older adults (Fiori et al., 2007). The quality of relationships with friends is determined by the quality of connection and the emotional distance between individuals: “Yes, relationships give meaning to life. To help them, (friends) to give them advice.” (I80, male, mid 70s); “If you’re friends with someone, you have to be a good friend, not a false one.” I64, female, mid 80s).

Older adults who maintain friendships tend to be more socially and psychologically well adjusted, benefiting from the emotional and instrumental support that these relationships provide (Adams & Blieszner, 1995). While larger networks can enhance life satisfaction, the quality and composition of these networks, particularly the presence of friends, play a crucial role in the well-being of older adults. Older adults prefer small groups of friends due to the immediate emotional benefits, opportunities for meaningful social engagement, and the supportive nature of these relationships: “[…] to make a small group, three or four people. I have made this group here […]. And we meet every day a quarter of an hour before breakfast, before lunch, another quarter of an hour.” (I70, male, early 80s).

Older adults can significantly benefit from social media friendships by enhancing social connections and reducing loneliness. The overall effectiveness of social media as a tool for improving well-being in older adults depends on individual circumstances and the nature of their online interactions: “I don’t get bored, (at home). I have friends on WhatsApp, Messenger.” (I86, male, mid 80s); “I was alone, … now I have (friends) if you believe me, on Facebook, a hundred of friends I have. And they all call me. There’s a community here.” (I74, male, early 70s).

Solitude in older adults can be both beneficial and detrimental, depending on whether it is voluntary or not, and it also depends on the quality of their social relationships. While solitude can enhance positive affect and provide personal time, it can also lead to loneliness and depression if not balanced with meaningful social interactions and valuable relationships (Ong et al., 2015).

Self-isolation and loneliness are significant for older adults, with implications for their physical and mental health. Addressing self-isolation and loneliness in older adults requires a multifaceted approach that considers individual, cultural, and contextual factors. These issues are prevalent among the aging population, particularly in the context of increasing life expectancy and societal changes: “You end up retreating, … like a turtle in its shell, and you sit and you think… maybe where I went wrong, maybe I did something wrong, right?” (I62, female, late 60s); “Most of the time I feel isolated. I mean not isolated, because no one isolates me, but… it’s hard not to have anyone close.” (I64, female, mid 80s); “Loneliness is very hard.” (I65, male, mid 80s); “My wife died June 16 last year. The truth is that it was very hard and still is to me. I live alone in my room, I have nobody close to me.” (I70, male, early 80s).

The death of a spouse in elderly adults leads to increased depression, anxiety, and cognitive decline, with social isolation being a significant risk (Hung et al., 2020). Interventions focusing on emotional support and social engagement can aid in mitigating these impacts: “After my husband died, I felt so lonely. I chose to stay alone, and after a while, after a year, I found out about this [retirement] home, and I thought it would be better to be somewhere, not to disturb my daughter.” (I63, female, early 70s).

Unsatisfactory social relationships can have significant negative impacts on the well-being of older adults. These impacts manifest in various aspects of mental and physical health, as well as overall life satisfaction (Santini et al., 2015). While older adults may develop resilience to some negative interactions, the overall impact of poor relationship quality remains significant, particularly for mental health: “Most of the (time) I stay isolated… no one isolates me, but… it’s hard to have someone close to you.” (I64, female, mid 80s); “You get criticized that it’s too much, … so, you end up retreating, just like a turtle in its shell and you [start] thinking [yourself] I did something wrong?” (I62, female, late 60s); “Relationships make sense. If you don’t relate to people, you’re lonely. You drift away. Loneliness kills.” (I81, male, mid 80s).

The importance of not being a burden for older adults is multifaceted, involving cultural, emotional, and practical dimensions. Older adults’ concerns about being a burden are shaped by their cultural background and emotional relationships with their children (Cahill et al., 2009). The concept of not wanting to be a burden was found to be significant among older adults, particularly in the context of their relationships with their children: “The children decided to put me in the nursing home.” (I70, male, early 80s); “I am here because my children relocated abroad, and I didn’t want to be a burden for the children.” (I71, female, late 70s).

### 4.4. Life Objectives

It is important to recognize the value of short-term and long-term objectives for older adults. These objectives can provide structure, enhance quality of life, and support successful aging. Short-term objectives, such as engaging in enjoyable activities and physical exercise, can provide immediate benefits and motivation for older adults (Devereux-Fitzgerald et al., 2016). Long-term objectives focus on maintaining health and cognitive function, supported by effective self-management and communication strategies (Lawless et al., 2021). Both types of objectives are essential for enhancing the quality of life and supporting successful aging in older adults.

By focusing on health (Travers et al., 2021), socialization and independence (Pfund et al., 2021), older adults may be able to maintain a sense of purpose and engagement, which are key to defining meaning in life. It has been suggested that there are several types of goals that are common to older adults: (i) goals related to purpose and engagement (Howard & Louvar, 2017); (ii) goals related to autonomy, health, relationships, and emotional comfort which are integral to older adults’ quality of life (Van Leeuwen et al., 2019); (iii) for older adults with long-term conditions, self-care and self-management objectives are essential (Lawless et al., 2021). In the current research, we identified two categories of objectives: daily objectives and short-term objectives

The following daily objectives (day-to-day functioning, religious practices, entertainment, purpose, and engagement) have been proposed by the members of the research group: “here (in the nursing home, my wife and I) have a schedule that I aim to be as exact as possible; after we get up in the morning and have breakfast, we go to the city center on foot, then at the church. We had lunch at noon, then we went to the art exhibitions.” (I66, male, early 70s); “In the morning, we worship God.” (I65, male, mid 80s).

Functional activities are essential for older adults as they promote physical health, enhance psychological well-being, and improve quality of life (Owen et al., 2022). These activities help maintain independence, manage chronic conditions, and reduce the risk of falls, making them a vital component of care for the elderly: “We go out to eat, come back, rest and that’s about it.” (I64, female, mid 80s); “In the morning, to do my chores, to have my clothes clean.” (I87, male, early 80s).

Instrumental activities of daily living are vital for the independence and well-being of older adults, influencing their health, social engagement, and overall quality of life. Effective interventions and an active lifestyle can enhance instrumental activities of daily living performance, while psychosocial factors and technological advancements also play significant roles in their lives: “I still go, and I still help my daughter on holidays, when she’s busy.” (I63, female, early 70s); “I have in my room, TV, laptop, I talk on WhatsApp and Messenger.” (I86, male, mid 80s).

Recreational activities play a vital role in promoting health, happiness, and social connectedness among older adults (Ryu & Heo, 2018). By providing opportunities for physical activity, social interaction, and purposeful engagement, these activities contribute significantly to the well-being and quality of life in aging populations: “I read a book, go to the club, play a Rummy, do tailoring.” (I62, female, late 60s); “Out for a walk.” (I67, female, late 60s); “I’d like to go to the seaside, to Slănic Moldova (mountain resort), where I’ve been (when I was younger).” (I87, male, early 80s).

Traveling in later life offers older adults a pathway to spiritual and emotional enrichment, social connection, and personal development. By addressing the challenges and barriers they face, we can support their journeys toward finding meaning and satisfaction in life (Moal-Ulvoas, 2017). Traveling is a common goal among older adults, often linked with finding a calling and mastering new skills. This pursuit not only provides enjoyment but also contributes to a sense of achievement and personal growth: “… in the summer, for example, I planned to go to Denmark, to my sons.” (I71, female, late 70s); “…to go and see Florence (where my son lives).” (I84, male, early 80s).

Considering their age, the elderly mentioned that they do not imagine and do not have long-term plans: “I like to live the moment, day by day, because you don’t know how far (life goes).” (I62, female, late 60s), or they have plans within a maximum time horizon of one year: “I have always had the plan to go somewhere, to go in summer, spring… I would like to go to Sovata… there is the swimming pool.” (I75, male, mid 80s); “… let’s go on a trip with our coach from here (from the nursing home).” (I85, female, mid 80s).

## 5. Discussions and Conclusions

The majority of the themes identified in the present research appear to corroborate findings from previous studies (e.g., North & Fiske, 2015), suggesting shared characteristics among elderly individuals that may transcend geographical and cultural boundaries.

However, our investigation group exhibited distinctive orientations, notably a more individualized and critical approach to religious practice, rather than strict adherence to institutionalized rituals, as well as a pronounced focus on moral transmission and personal legacy as primary sources of meaning. These particularities likely reflect the socio-cultural context and historical experiences specific to the studied region, shaping unique meaning-making pathways among the elderly participants.

For a considerable number of old individuals, religiosity is defined as the extent of religious belief and practice in an individual’s life, and it has been historically regarded as a fundamental source of meaning. Its role as a cornerstone of meaning can be analyzed as a link to transcendence, as it often provides individuals with a connection to something greater than themselves, whether it is God, a divine principle, or the cosmic order.

In the group of older adults who were the focus of the study, the qualitative analysis yielded four sources of meaning, as outlined below: (i) religiosity/spirituality, (ii) the relations with others, (iii) the moral principles that govern life, and (iv) specific life goals.

The analysis of the frequency of statements pertaining to “God”, “Divinity”, “Universe”, and “Higher force” has identified religiosity/spirituality as a primary source of meaning, one that is more or less connected to the other sources of meaning that emerged from this study. This phenomenon can be attributed to two factors. First, the frequency with which this theme emerged in the interviews exceeded that of other themes. Second, this theme was a recurring topic that manifested across the entire discourse of the interviewed individuals, irrespective of the subject under discussion. There are several directions and conclusions that can be identified as elements of novelty brought by the current research.

One noticeable conclusion is the way religiosity is seen in various forms:

- as a matter of religious conviction;
- as a matter of religious practice;
- as content in the process of intergenerational transmission;
- as a value in itself;
- as a grid for reinterpreting reality;
- as a filter for selecting choices/decisions;
- as a foundational element in the grounding of the meaning of life.

As evidenced in previous research (Abeyta & Routledge, 2018; AbdAleati et al., 2016), religiosity manifests in various forms, and it has been demonstrated to exert influence on mental health, life satisfaction, and social relationships.

We have to underline that the interpretation of religiosity and spirituality by older people is unique in its own right. Important ideas that are part of the mainstream of religion, such as the punitive or rewarding character of life after death and the influence of destiny by divinity, take particular forms in this context (e.g., the non-acceptance of the existence of life after death, implacable destiny are not sources of meaning for older adults). These forms are transferred to the current life (fulfilled or unfulfilled life, the meaning of suffering, etc.), observations confirmed by previous research (Zimmer et al., 2016).

Another significant observation, which is in line with previous studies (Chamberlain and Zika, 1988), is that a substantial number of the values expressed by the subjects in the study seem to be influenced by previous religious traditions and practices. This assertion is supported by the fact that religious practices and beliefs offer a sense of purpose and direction, thereby providing psychological support. Another observation is that the presence of a community that shares similar religious beliefs and intentions has the potential to foster a sense of social cohesion, thereby offering additional psychological comfort. Finally, another observation is linked to the notion of predetermined destiny, influenced by a divine entity, that provides elderly people with a sense of purpose and induces the idea that it is a contribution to a larger plan. This plan can pertain to the evolution of society as a whole or to a divine plan that is believed to promote unanimity.

The second source of meaning in life identified by the research group, which refers to interpersonal relationships, has the potential to endow the lives of elderly individuals with significance and foster well-being. Some believe that as people age, the importance of relationships may increase, enhancing their sense of coherence and continuity in life (Schnell, 2009). It has been observed that elderly individuals appear to be aware of the significant role that relationships may play in providing meaning and well-being (Adams & Blieszner, 1995).

In these circumstances, while relationships with friends are typically regarded as a positive source of emotional meaning (O’Connor, 1995), we can observe that family relationships are considered both positive and negative emotional sources. It has been acknowledged in the literature that family relationships can have a positive impact on the elderly. However, the impact of dysfunctional family relationships on the elderly has received less attention in research. Within the frame of the current research, one can observe that this phenomenon has the capacity to modify the behavioral profile of the elderly regarding their own family.

In the research group, it was observed that in cases where the relationship is deemed dysfunctional, the elderly person may endeavor to adapt to the family dynamics, which can prove to be a substantial source of stress. Older adults’ perceptions of their roles within the family are considered to be a multilayered topic that involves understanding their contributions, the dynamics of family relationships, and social attitudes toward aging.

When the elderly person perceives their relationship with their family as a source of burden for their children, they opt for self-isolation, withdrawing from their family within the nursing home. A significant number of elderly individuals have chosen to relocate to a nursing facility due to their children’s international migration.

The third significant source of meaning that was identified in the current research refers to the moral principles, which encompass attitudes and instruments of behavioral regulation. It can be postulated that religion, as stated above, serves as the predominant source of moral values and behavioral principles. In this paradigm, the responsibility for educating younger generations and for the transmission of life principles by elders is viewed as occurring within the broader context of religious belief systems.

Within this theme that emerged from the interviews, we found the notion of *intergenerational transmission of fundamental values and principles* that govern human existence as a whole. It is largely accepted that intergenerational transmission of values is a complex process influenced by familial, cultural, and social factors (Schönpflug, 2001). It plays a crucial role in cultural continuity and the socialization of future generations, and in this context, several broad categories were identified; the respondents believe that these should be transmitted to younger generations as an expression of life experience. The identified categories encompass: *faith as such* (religious values and principles), *general human values* (which may align with those proposed by religion), and *professional values* (which appear to be independent of religious faith).

Based on the current research, *the process of intergenerational transmission of values and principles* can be regarded as a generator of meaning in itself. One of the most important conclusions from our study is that the elderly population perceives this transmission as a moral obligation, intrinsically linked to their life stage and their status as elders within the family structure, particularly with regard to their grandchildren. Within the paradigm of intergenerational transmission, religious practices assume the role of both an instrument and a means, contributing to the perpetuation of religious and spiritual faith. In this context, the religious practices function as an external source of meaning, serving, on one hand, as a tool for educating the young and, on the other hand, as a resource to fortify the internal source of meaning embodied by religion.

The life objectives of the elderly population constitute another substantial source of meaning in life (Hupkens et al., 2018). These objectives are oriented toward daily activities related to functionality and autonomy (Pirhonen & Pietilä, 2015). However, some goals, particularly those of a recreational nature, are associated with family or friends and are designed for a period of time ranging from several months to a maximum of one year.

In contrast to other age demographics, we observed that when we queried the members of the research group about their long-term aspirations, they frequently asserted the absence of long-term goals. We explained this phenomenon in two ways. Firstly, it can be attributed to the awareness of advanced age and impending death. Secondly, it can be explained by the uncertainties related to their fragile physical state. A significant number of elderly individuals have been observed to articulate objectives that are oriented toward the present moment and the pursuit of enjoyment in life. This phenomenon, characterized by the deliberate savoring of life’s pleasures (Smith & Hollinger-Smith, 2015), is particularly notable within the context of this specific age demographic.

Apart from the four main sources of meaning that were identified within the research group, some particular topics appear to present certain contradictions. One of these topics is the discourse surrounding older adults’ matter of “life after death”. It is interesting to note that, despite the strong religious beliefs expressed by the study participants, their views on life after death vary greatly. Although religious perceptions propose and encourage behaviors and attitudes from a rewarding or punitive perspective in the afterlife, it was expected that individuals with a strong religious belief would find motivation for certain behaviors and attitudes, having by default a source of meaning in the idea of life after death. However, the opinions regarding life after death do not necessarily seem to relate to religious beliefs. Also, a large number of respondents stated that they do not believe in life after death, despite their strong religious belief: “I believe in God. I don’t believe in life after death.” (I71, female, late 70s); “I can’t say that it (life after death) exists.” (I64, female, mid 80s); “It could be (life after death).” (I65, male, mid 80s); “It exists (life after death). I think hell is on earth here.” (I62, female, late 60s); “It’s a continuation of present life.” (I63, female, early 70s); “I don’t believe in life after death (as the orthodox religion describes it). ”I believe in reincarnation.” (I66, male, early 70s).

Another marginal topic was found in a negligible proportion of the participants self-identified as atheists, attributing human existence to Darwinist evolutionism: “Fear of (life after) death has not affected my decisions in life, as long as I live here, I do what I think is right, not to affect others, to be good, with family and friends.” (I63, female, early 70s). This assertion is supported by the observation that “life choices and actions in life are not influenced by the idea of death, but rather by the idea of life” (I66, male, early 70s). In this context of analysis, the concept of life after death does not inherently signify a source of meaning.

In the end, we can state that the existence of all these sources of meaning does not guarantee a state of well-being or a high quality of life. However, it appears to provide the necessary intentional coherence, acceptance of reality, and access to coping mechanisms, whose adaptive nature depends on the rigidity of thinking of old people. Further research is necessary to fully elucidate the implications of this phenomenon.

While this study provides valuable insights into the sources of meaning for institutionalized elderly adults, future research should expand the sample to include non-institutionalized and culturally diverse populations to improve generalizability. Additionally, a mixed-methods approach (combining qualitative depth with quantitative validation) could further verify thematic patterns. Exploring the interplay between religiosity, intergenerational transmission, and social relationships may reveal deeper mechanisms by which older adults construct meaning. Such investigations could inform more tailored interventions to enhance well-being in aging populations.

Although broadly consistent with international findings, the results of this study highlight culturally specific orientations among Romanian institutionalized elderly individuals, including a critical and personalized approach to religious practice and a pronounced emphasis on intergenerational moral legacy. These findings underline the necessity of considering cultural and socio-historical contexts when interpreting meaning-making processes in older adulthood.

This study highlights key areas that could guide interventions aimed at enhancing meaning in life among institutionalized elderly residents. Encouraging opportunities for spiritual engagement, promoting intergenerational value transmission, fostering social connectedness, and supporting personal goal-setting could strengthen residents’ sense of purpose and fulfillment. By addressing these dimensions, retirement homes may significantly improve the psychological well-being and overall quality of life of their residents.

## Data Availability

All data related to this article are available upon request (in the Romanian language).

## Appendix A. COREQ Checklist

The following table summarizes the reporting of the applicable items from the COREQ 32-item checklist for this study.

Domain 1: Research team and reflexivity	
**Item**	**Description in the Study**
Research team	Interviews were conducted by two doctoral students and a coordinating professor.
Education and occupation	All researchers had higher education in psychology, and prior experience in qualitative research.
Relationship with participants	No prior relationship with participants; recruitment was voluntary after study objectives were explained.
Personal relevance and reflexivity	Researchers used reflexivity techniques to minimize personal bias.

Domain 2: Study design	
**Item**	**Description in the Study**
Setting	Interviews were conducted at “Saint Pious Parascheva” nursing home, in private dedicated spaces.
Participants	25 individuals aged between 67 and 93 years; inclusion criterion: institutionalized residents.
Recruitment	Voluntary recruitment after presenting study objectives.
Interview guide	Semi-structured, based on specialist literature; informally pre-tested.
Data collection	Audio-recorded interviews with explicit consent; approx. 60 min each.
Data saturation	Thematic saturation reached after 20 interviews; remaining interviews confirmed findings.

Domain 3: Analysis and findings	
**Item**	**Description in the Study**
Transcription	Full transcription with Transkriptor© software; manually validated by researchers.
Software used	NVivo© used for initial coding; manual processing later due to language limitations.
Method of analysis	Inductive thematic analysis; coding triangulation between researchers.
Validity and rigor	Continuous reflexivity; consensus on main themes.
Confidentiality	Anonymization through coded identifiers; secured storage of audio and text data.
